# Parenting Practices and Adolescents’ Mental Health: The Mediating Role of Perceived Maternal and Paternal Acceptance-Rejection and Adolescents’ Self-Efficacy

**DOI:** 10.3390/ijerph20021052

**Published:** 2023-01-06

**Authors:** Shin Ling Wu, Pei Jun Woo, Chin Choo Yap, Glen Johan Ri Young Lim

**Affiliations:** Department of Psychology, School of Medical and Life Sciences, Sunway University, Petaling Jaya 47500, Malaysia

**Keywords:** parental practices, parental acceptance-rejection, self-efficacy, adolescents, mental health

## Abstract

Parenting practices are essential in promoting children’s mental health, especially in effective and ineffective parenting. The use of ineffective parenting practices is no longer encouraged in the west; however, it remains a common practice among Asian households. Ineffective parenting consists of inconsistent discipline, corporal punishment, and poor monitoring which may result in mental health consequences. Thus, this study assessed the mediating effects of adolescents’ self-efficacy and parental acceptance-rejection on the relationship between ineffective parenting practices and adolescents’ mental health. The current study involved a total of 761 school-going Malaysian adolescents aged 13–18 (38.5% males; M_age_ = 15.65; SD_age_ = 1.43). This study utilized a cross-sectional design where it measured adolescents’ mental health, ineffective parenting practices, parental acceptance-rejection, and adolescents’ self-efficacy. Both paternal and maternal parenting practices and acceptance-rejection were measured independently. Adolescents’ self-efficacy and perceived paternal and maternal acceptance-rejection were found to be significant mediators for ineffective parenting practices and adolescents’ mental health. Our findings suggest that ineffective parenting practices will result in perceived parental rejection and lower self-efficacy which in turn resulted in poorer mental health among adolescents. It means parents should be mindful of their parenting approaches as they have a direct and indirect impact on the mental health of their offspring.

## 1. Introduction

It is estimated that 10–20% of adolescents in the world today suffer from some form of mental illness [1], where the negative state of mental health is also evident with suicide as the fourth leading cause of death among 15–19-year-olds [2]. The state of mental health in Malaysia is no better, as several studies recorded a high prevalence of suicidal ideation, indicating poor mental health among Malaysian adolescents [3,4]. Aside from emotional problems, poor mental health may have long-term consequences for adolescents such as self-harm, violent tendencies, and suicidal ideation [3,4,5]. Thus, the mental health of adolescents is a critical issue that should be addressed.

Adolescence is a critical period of development with heightened stress due to overwhelming changes [6]. There are many factors that contribute to adolescents’ mental health and well-being. Among them are stressful life events [7], parent-child conflicts [8], mistreatment by parents [9], and peer influence [10]. Despite the fact that there are numerous factors associated with mental health issues, it is generally recognized that the family environment plays a foundational role in the development of mentally healthy adolescents [8,11]. In other words, parental factors such as the type of parenting practice and the quality of the parent-child relationship set the stage for the development of mental health in adolescents [12,13].

Parents influence the mental health of their children through the use of parenting practices, which are forms of parent-child interactions that directly influence the outcomes of the child. Furthermore, Pascual-Sanchez and colleagues [9] found that ineffective parenting practices (IPP), which is defined as the parental use of poor monitoring, inconsistent discipline, and corporal punishment seem to be risk factors for adolescents’ mental health, while effective parenting practices (EPP) were protective factors. Parental warmth was found to promote better psychological adjustments [14], empathy, and self-concept among Spanish adolescents [15]. In a study conducted in Chile and Ecuador, IPP such as corporal punishment and poor monitoring predicted lower self-efficacy and higher frequency of externalized behavior [16]. In the same study, EPP such as positive parenting was associated with better self-efficacy and psychological well-being [16]. There were also similar findings in other studies, where IPP were linked with poorer adolescents’ mental health [9,11,17].

Although there is vast evidence of the negative effects of ineffective parenting practices, IPP like corporal punishment remains widely used by other ethnic groups such as African American [18], Arab [19], and Asian parents [20,21]. This could be because, while the link between IPP and poor adolescents’ mental health is widely found in Western families [9,16], research from the East [20,22,23] and other ethnic groups [18,24] appears to yield contradictory results. For example, Chao [24] found that authoritative parenting benefits European American adolescents while authoritarian parenting is more beneficial to Asian American adolescents. Meanwhile, Dwairy et al. [19] found that authoritarian parenting is not detrimental in Arab society. In terms of harsh physical punishment, African American children who experience harsh physical punishment were found to be less aggressive and have lower externalizing behaviors while European American children displayed higher aggression and externalizing behavior when harsh physical discipline is used [18]. This could be explained in terms of the living contexts whereby authoritarian parenting may be beneficial when the living environment is dangerous as it serves as a protective measure for the children [25].

In Asia, guan parenting is an Asian parenting construct in which parents place a great emphasis on strictness and a high level of concern in guiding their children [26]. Ang and Sin [27] found that Asian adults feel more secure and less anxious when receiving more guan parenting. Moreover, when guan parenting is used, Asian adults are better at emotional regulation and have higher self-esteem [27]. In Malaysia, guan parenting was found to correlate positively with adolescents’ development whereby adolescents reported higher life satisfaction when parents use guan parenting [28]. Chong and Yeo [20] found Malaysian young adults to be psychologically well-adjusted even though they experienced IPP such as corporal punishment during childhood. Such contradictions in findings could be because Malaysian adolescents themselves perceive IPP (i.e., corporal punishment) as a form of parental care [29]. These studies challenge the current criticisms of IPP by highlighting the impact of cultural differences on adolescents’ perspectives.

In an attempt to understand the importance of adolescents’ perceptions, Rohner [5] conceptualized the interpersonal acceptance-rejection theory (IPARTheory). According to IPARTheory, children are the active recipients of parental behaviors and how a child interprets that behavior will shape their outcomes. Thus, children who perceive themselves to be accepted (being cared for or loved by a parent) are likely to develop positive mental health. In contrast, they may develop mental health issues if they feel rejected by their parents [30,31,32]. A past study found that the impact of corporal punishment on youth’s psychological adjustment was mediated by children’s perceptions of parental rejection [33]. Qu and colleagues [34] further highlighted the importance of perceived parental acceptance-rejection where they found that when adolescents perceived maternal IPP such as poor monitoring as a form of maternal rejection, they will then develop mental health issues. This could be explained using the cross-cultural differential effect where parenting practices were perceived differently by children from different cultures. Chao [22] explained that parental control and strictness are not viewed negatively by Chinese children but are perceived as a form of parental care and concern. Therefore, Asian adolescents may interpret IPP as parental acceptance. If they do, IPP such as poor monitoring or corporal punishment may be less likely to result in mental illness among adolescents.

Another factor that plays a role in adolescents’ mental health is self-efficacy. Bandura’s Social Cognitive Theory defined self-efficacy as the belief in one’s capability to succeed in a specific situation or task [35]. According to the theory, self-efficacy plays an important role in the regulation of emotional states and mental health in general because self-efficacy determines the capability of an individual to regulate the daily stressors of life [36]. The importance of self-efficacy is supported by Schönfeld and colleagues [37], who found that lower self-efficacy was associated with more psychological problems, as individuals with low self-efficacy did not believe that they have the capabilities necessary to face challenging situations.

The development of self-efficacy is influenced by our social environment. That means peers, school, and family play an important role in the development of adolescents’ self-efficacy [35]. Past studies found that parents are particularly important in developing self-efficacy beliefs in their children [36,38]. In a study by Shen [39], it was found that self-efficacy was negatively influenced by IPP, as adolescents who received IPP such as corporal punishment believed that they have less agency over their outcomes. Thus, the researchers hypothesize that self-efficacy could be a mediator in the relationship between IPP and adolescents’ mental health.

In terms of mental health and parenting practices, there are mixed findings in terms of the contributions from fathers and mothers [40,41]. Some studies found that both parents employed similar amounts of IPP [39,41] while other studies showed that fathers employed more IPP [20,40]. There were also discrepancies in the influence of fathers and mothers on adolescents’ mental health. For instance, Sultana and Khaleque [32] found that adolescents’ psychological adjustment was more affected by paternal IPP as compared to the maternal IPP. On the other hand, a couple of studies suggested that both fathers’ and mothers’ IPP had a similar influence on the mental health of their adolescents [20,41]. Moreover, Rinaldi and Howe [42] suggested that different parenting practices by mothers and fathers contributed differently to adolescents externalizing and adaptive behaviors. Thus, our study aims to understand both father and mother parenting practices independently.

The current study sought to understand the possible mediating effects of self-efficacy and parental acceptance-rejection on the relationship between ineffective parenting practices (IPP) and adolescents’ mental health. IPP of both fathers and mothers were measured independently.

## 2. Materials and Methods

### 2.1. Study Design

This study utilized a quantitative cross-sectional research design. Cross-sectional research design was used as it is able to assess the mental health status of a given population at a single point in time. Adolescents are the studied population where the predicting variable is ineffective parenting practices, the outcome variable is adolescents’ mental health, and the mediating variables are adolescents’ self-efficacy and adolescents’ perception of parental acceptance/rejection.

### 2.2. Participants

Purposive sampling method was used to recruit the participants. A minimum sample size of 691 was projected using a priori power via G*Power with an alpha = 0.05, power = 0.95, and small effect size = 0.025 in a F-test. An additional 20% was added to the projected number to account for attrition. Finally, the study consisted of 761 Malaysian school-going adolescents aged 13–18 (M_age_ = 15.65; SD_age_ = 1.43). The participants in our study consisted of 468 females (61.5%) and 293 males (38.5%). The ethnicity of the participants reflects the ethnicity ratio of Malaysia with 422 (55.5%) Malays, 266 (34.9%) Chinese, 52 (6.8%) Indians, and 21 (2.8%) adolescents from other ethnic backgrounds.

### 2.3. Measures

Adolescents’ Mental Health. Mental health was measured using the Strengths and Difficulties Questionnaire (SDQ) [43], completed by the adolescents. The SDQ is a 25-item scale with 5 subscales. As recommended by the author, only 20 items from 4 subscales namely–conduct problems, hyperactivity, emotional problems, and peer problems were summed up to represent adolescents’ mental health. Sample items include “I am often accused of lying or cheating.” for conduct problems subscale, “I am restless, I cannot stay still for long.” for hyperactivity subscale, “I am often unhappy, depressed or tearful.” for emotional problems subscales, and “Other children or young people pick on me or bully me.” for peer problems subscale. All four subscales contribute positively to the total scale score, where a higher score means more mental health issues. Idris and colleagues [44] found that the scale had good concurrent validity. Our analysis found that the Cronbach’s alpha of the scale was α = 0.79.

Perceived Parental Acceptance-rejection. Adolescents’ perception of parental acceptance-rejection was measured using the total score of the Parental Acceptance-rejection Questionnaire for children which consisted of 24 items [45]. A self-report measure was selected because Rohner [5] believes that it is the perspective of the child that is important in studying the mental health of the child. This scale consists of four subscales namely warmth, hostility, indifference, and undifferentiated rejection which were totaled to represent perceived parental acceptance-rejection. All 24 items were measured on a 4-pointed Likert-type scale that ranges from ‘Almost Never True’ (1) to ‘Almost Always True’ (4). Sample item for warmth subscale is “My parents say nice things about me”; sample item for hostility subscale is “My parents made me feel unloved if I misbehaved”; sample item for indifference subscale is “My parents made it easy for me to tell them things that were important to me”; and sample item for undifferentiated rejection is “My parents paid no attention when I asked for help”. The warmth subscale contributes negatively to the total scale score while hostility, indifference, and undifferentiated rejection contribute positively to the total scale score. Thus, all items in the warmth subscale were reversed scored. A higher total score indicated higher parental rejection. Convergent and discriminant validity was established for the Parental Acceptance-rejection Questionnaire [45]. In this study, the internal consistency of the scale was α = 0.90 for both fathers and mothers.

Ineffective Parenting Practices (IPP). IPP was measured using the Alabama Parenting Questionnaire [46]. The Alabama Parenting Questionnaire is a 42-item self-reported questionnaire that assesses parenting practices. The scale has 5 subscales, namely Inconsistent Discipline (6 items), Positive Parenting (6 items), Involvement (10 items), Poor Monitoring (10 items), Corporal Punishment (3 items), and other disciplinary methods (7 items). The total scores of inconsistent discipline, poor monitoring, and corporal punishment were summed up to obtain an ineffective parenting score. All three subscales contribute positively to the total scale score where a higher total score indicated more IPP. Sample items include “Your parents threaten to punish you and then do not do it” for inconsistent discipline subscale, “Your parents do not know the friends you are with” for poor monitoring subscale, and “Your parents spank you with their hand when you have done something wrong” for corporal punishment subscale. Discriminant validity of the questionnaire was established [47,48]. The Cronbach’s alpha coefficients for the IPP in this study were α = 0.80 and α = 0.90 for fathers and mothers, respectively.

Adolescents’ Self-efficacy. Adolescents’ self-efficacy was measured using the General Self-Efficacy Scale [49]. Self-efficacy was calculated using the total score of the 10 self-reported items on the scale. Each item was measured on a 4-point Likert-type scale that ranged from ‘Not True at All’ (1) to ‘Exactly True’ (4). A higher total score indicated higher self-efficacy. Concurrent and predictive validity were established for this scale [49,50]. Cronbach’s alpha coefficient for the scale was α = 0.91 in this study.

### 2.4. Data Collection Procedure

Ethical clearance for this study was given by the Institute Ethics Committee. Approvals from the Ministry of Education of Malaysia, States Education Department, and parents were also obtained as the researchers collected data from high school students in government high schools. Data collection was conducted from August 2021 to December 2021. Face-to-face and online methods were used for data collection as most of the classes were held online due to the COVID-19 pandemic. For face-to-face data collection, hardcopies of the questionnaires were distributed to the participants with the help of the schoolteachers. Whereas for online data collection, parents were first approached online to obtain their approval. Parents who consented would then pass the Google Form to their children. The Malay version of the scales were used in both methods.

### 2.5. Statistical Analysis

SPSS version 27 was used to analyze the data. The correlation between all the study variables was first examined using Pearson’s correlation analysis. Next, a parallel mediation model was analyzed using the PROCESS Model 4 by Preacher and Hayes [51] to explore the mediating effects of self-efficacy and parental acceptance-rejection on the relationship between IPP and adolescents’ mental health. For bootstrapping, we used 5000 samples for the current analyses with a confidence interval of 95%. The mediation models of paternal and maternal ineffective parenting were tested independently.

## 3. Results

### Descriptive Information

Table 1 displays the descriptive information for parental acceptance-rejection and adolescents’ mental health. The majority of the participants reported their fathers to be accepting (78%), while a minority of 62 participants (8.1%) reported having rejecting fathers. For maternal acceptance-rejection, 644 (84.6%) participants perceived their mothers to be accepting while 47 (6.2%) participants perceived their mothers to reject them [52,53]. Based on the cut-off point in the National Health and Morbidity Survey [54], participants in our study were mostly mentally healthy with 62.3% of our participants having little mental health issues. However, 37.7% of participants had abnormal levels of mental health problems.

Table 2 shows the means, standard deviation, and inter-correlations of the variables in the study. Based on Table 2, paternal ineffective parenting practices (IPP) were negatively correlated with adolescents’ self-efficacy (*r* = −0.14, *p* < 0.001), and positively correlated with paternal acceptance-rejection (*r* = 0.45, *p* < 0.001) and adolescents’ mental health (*r* = 0.40, *p* < 0.001). Maternal IPP was also negatively correlated with adolescents’ self-efficacy (*r* = −0.13, *p* = 0.001), and positively correlated with maternal acceptance-rejection (*r* = 0.46, *p* < 0.001) and adolescents’ mental health (*r* = 0.41, *p* < 0.001). Results show that the more IPP used by mothers and fathers, the lower adolescents’ self-efficacy, the higher the perceived parental rejection, and the higher the likelihood of mental health problems among adolescents.

**Figure 1:**
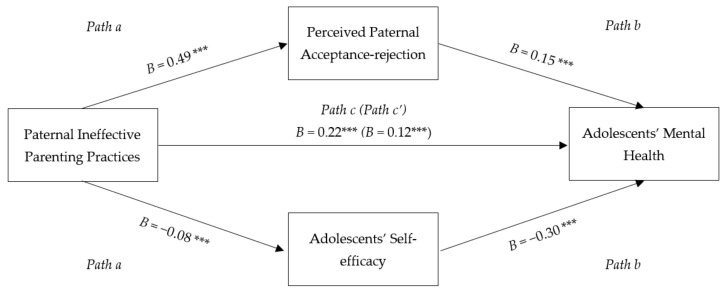
Mediation Model of the Relationship between Paternal Ineffective Parenting Practices and Adolescents’ Mental Health. Note. Coefficients presented are unstandardized regression coefficients. *** *p* < 0.001.

Figure 1 illustrates a parallel mediation model conducted using PROCESS Model 4 [55]. Based on the results, higher usage of paternal IPP was linked to higher paternal rejection, which then was associated with more adolescents’ mental health problems (indirect effect point estimate = 0.08, *SE* = 0.01, 95% Cl [0.06, 0.10]). That means more paternal IPP was associated with more perceived paternal rejection, which then influence an increase in mental health problems. The results also showed that higher usage of paternal IPP was associated with decreased self-efficacy, which was in turn linked to poorer adolescents’ mental health (indirect effect point estimate = 0.02, *SE* = 0.01, 95% Cl [0.01, 0.04]). It can be concluded that perceived paternal acceptance-rejection and adolescents’ self-efficacy mediated the relationship between paternal IPP and adolescents’ mental health.

**Figure 2:**
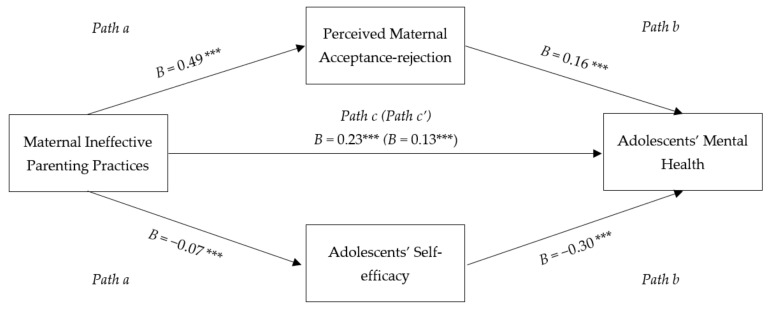
Mediation Model of the Relationship between Maternal Ineffective Parenting Practices and Adolescents’ Mental Health. Note. Coefficients presented are unstandardized regression coefficients. *** *p* < 0.001.

The results were similar for maternal ineffective parenting. Based on Figure 2, the results showed that maternal IPP correlated with perceived maternal acceptance-rejection, which was then associated with adolescents’ mental health issues (indirect effect point estimate = 0.08, *SE* = 0.01, 95% Cl [0.06, 0.10]). That means more maternal IPP usage was linked with more perceived maternal rejection, which then will increase mental health problems in adolescents. Higher usage of maternal IPP was also associated with lower adolescents’ self-efficacy, which in turn was linked to poorer adolescents’ mental health (indirect effect point estimate = 0.02, *SE* = 0.01, 95% Cl [0.01, 0.04]). Hence, perceived maternal acceptance-rejection and adolescents’ self-efficacy were significant mediators on the relationship between maternal IPP and adolescents’ mental health.

## 4. Discussion

The primary purpose of our study is to understand the extent to which perceived paternal and maternal acceptance-rejection and adolescents’ self-efficacy mediate the relationship between ineffective parenting practices (IPP) and adolescents’ mental health. In our study, IPP are defined as the use of poor monitoring, inconsistent discipline, and corporal punishment. The present study found that IPP has a significant link with adolescents’ mental health, which is consistent with past studies whereby IPP affect children’s development [20,23,56,57,58,59,60]. The multiple mediators model also revealed that adolescents’ perceived parental acceptance-rejection and adolescents’ self-efficacy are mediators for the relationship between IPP and adolescents’ mental health. Specifically, adolescents who experienced more IPP were more likely to perceive themselves to be rejected by their parents as well as develop lower self-efficacy, which then leads to more mental health complications.

The current findings identified perceived parental acceptance-rejection as a significant mediator in the relationship between IPP and adolescents’ mental health. That means adolescents with parents who use IPP may perceive a sense of rejection by their parents, which is then linked to poor mental health among adolescents. This is consistent with past findings which found that IPP were linked with a sense of parental rejection, lowering their mental health [32,34,61]. The findings of the present research provide support for the IPARTheory, which postulates that perceived parental acceptance-rejection plays a major role in the emotional, behavioral, cognitive, and social development in children [9,62,63,64,65]. As explained by Rohner [5], this is because humans have a need for positive response, including an emotional desire for comfort, care, and love (otherwise known as parental acceptance). When parents meet their offsprings’ emotional needs, their development will go well. However, when parents have a tendency of using IPP such as poor monitoring, adolescents may perceive the lack of supervision as a form of parental rejection. Since there is an incongruence between adolescents’ need for love and acceptance, and adolescents’ perceived sense of parental acceptance-rejection, adolescents may feel emotionally deprived, leading to psychological maladjustment. Therefore, the qualities of parenting practices have a strong impact on adolescents’ sense of well-being because of how adolescents may perceive it.

Our findings expanded past research on the role of self-efficacy on mental health [35,37,66,67,68] whereby we found that IPP have a detrimental influence on self-efficacy, which were in turn, linked to poorer adolescents’ mental health. This is consistent with past studies that discovered IPP, such as poor monitoring and use of corporal punishment, to be linked with lower self-efficacy, and subsequently poorer adolescents’ mental health [38,39]. According to the Social Cognitive Theory, adolescents depend on their environment and the people around them to develop a sense of self-efficacy [35]. Hence, when parents (who are the key socializing agents in their lives) employ IPP such as corporal punishment, adolescents’ self-efficacy may be threatened, making them less likely to believe in their own agency and competency. When adolescents see themselves as inadequate, they may judge themselves as incapable of controlling their own lives. To develop self-efficacy, Bandura [35] argued that parents should encourage their children positively and ensure that they are aware of their children’s activities to provide a sense of support and validation. However, parents who employ IPP such as poor monitoring do not fulfil these criteria, thus lowering the chances of developing self-efficacy among their children. In short, high usage of IPP is linked to lower self-efficacy, which is associated with more adolescents’ mental health problems.

In our study, both paternal and maternal parenting practices have significant influences on adolescents’ mental health. Present findings are aligned with past findings that acknowledge the importance of both parents in raising a mentally healthy adolescent [12,16,69,70,71,72,73]. Contradictory to some studies where it was suggested that fathers employed more IPP as compared to mothers [20,40], our data showed that both fathers and mothers seem to display similar levels of IPP. The similar usage of IPP could be due to fathers and mothers choosing partners that have similar characteristics and thoughts on parenting [74]. Seeing as to how influential both paternal and maternal IPP are on adolescents’ mental health, both fathers and mothers should be mindful of the parenting practices that they use.

This study has several indispensable strengths, such as a good distribution of the sample. The demographics of our participants were representative of Malaysian citizens, whereby our participants consisted mainly of Malays, followed by Chinese, Indians, and other races. Whereas our study studied parenting and adolescents’ mental health in a non-Western sample, most other parenting research were performed on Western samples. By doing so, we were able to research IPP, parental acceptance-rejection, and adolescents’ mental health from a Southeast Asian perspective.

Although our findings provide useful information, our study has several limitations that should be acknowledged. First and foremost, our study did not take into account parental harshness when collecting data on IPP. Future studies should take into account parental harshness when studying IPP to better understand the mechanism in which IPP is associated with adolescents’ mental health. The next limitation of our study is that our study only took into account the perspective of adolescents when studying parenting practices. Doing so may lower the objectivity of the study, as only adolescents’ views of parents is taken into account. Future studies should collect data from additional parties such as parents. Lastly, this study used a cross-sectional research design, thus we are unable to determine the causality of the variables. Future studies could conduct a longitudinal study to understand the cause and effect of parenting practices on adolescents’ mental health.

This study suggests that the Malaysian government and related NGOs could come up with programmes that educate parents about the consequences of ineffective parenting practices. Moreover, parenting programmes should also educate parents on the generational differences that exist between them and their children. This is because what parents are brought up with may differ greatly from the culture in which their children will grow in. Lastly, when considering which parenting practice to use, parents and relevant parties should consider the perception of the receiver. For example, parents should prioritize using parenting practices that would be perceived as a form of parental acceptance.

## 5. Conclusions

In conclusion, our study adds further evidence that highlights the link between ineffective parenting practices (IPP), adolescents’ self-efficacy, perceived parental acceptance-rejection, and adolescents’ mental health. Specifically, the study found the mediating role of adolescents’ self-efficacy and perceived parental acceptance-rejection on the relationship between IPP and adolescents’ mental health. It is suggested that IPP has adverse effects on children’s adjustment, and it may be important for parents to avoid IPP. Moreover, governments and non-governmental organizations could provide intervention programs that are aimed at educating parents on suitable parenting practices.

## Figures and Tables

**Table 1:** Descriptive Statistics of the Study Variables (N = 761).

Variables	*n*	%
Perceived Paternal Acceptance-Rejection		
Unrealistic Idealization (24–28)	88	11.6%
Perceived Acceptance (29–51)	505	66.4%
Moderate (52–59)	106	13.9%
Perceived Rejection (60–96)	62	8.1%
Perceived Maternal Acceptance-Rejection		
Unrealistic Idealization (24–28)	130	17.1%
Perceived Acceptance (29–51)	514	67.5%
Moderate (52–59)	70	9.2%
Perceived Rejection (60–96)	47	6.2%
Mental Health Issues		
Close to average (0–14)	474	62.3%
Abnormal (15–40)	287	37.7%

**Table 2:** Means, standard deviations, and correlations between the variables of the study.

Variables	Mean	SD	1	2	3	4	5	6
Paternal IPP	39.78	10.26	-					
Maternal IPP	39.59	10.10	0.89 ***	-				
Self-efficacy	30.03	5.94	−0.14 ***	−0.13 ***	-			
PARQ-P	41.50	11.02	0.45 ***	0.42 ***	−0.41 ***	-		
PARQ-M	39.16	10.76	0.42 ***	0.46 ***	−0.40 ***	0.77 ***	-	
Mental health	13.02	5.67	0.40 ***	0.41 ***	−0.46 ***	0.53 ***	0.54 ***	-

Note. *** *p* < 0.001. Paternal IPP, Perceived paternal ineffective parenting practices; Maternal IPP, Perceived maternal ineffective parenting practices; PARQ-P, Paternal acceptance-rejection; PARQ-M, Maternal acceptance-rejection. Higher scores on IPP, self-efficacy, PARQ, and mental health mean higher usage of perceived ineffective parenting practices, higher self-efficacy, higher perceived rejection, and higher mental health issues, respectively.

## Data Availability

The data that support the findings of this study are available on request from the corresponding author. The data are not publicly available due to privacy or ethical restrictions.
